# Complex Characterization and Behavior of Waste Fired Brick Powder-Portland Cement System

**DOI:** 10.3390/ma12101650

**Published:** 2019-05-21

**Authors:** Viviana Fátima Rahhal, Mónica Adriana Trezza, Alejandra Tironi, Claudia Cristina Castellano, Milena Pavlíková, Jaroslav Pokorný, Edgardo Fabian Irassar, Ondřej Jankovský, Zbyšek Pavlík

**Affiliations:** 1 Departamento de Ingeniería Civil Facultad de Ingeniería, Universidad Nacional del Centro de la Provincia de Buenos Aires, Av. del Valle 5737, B7400JWI Olavarría, Argentina; vrahhal@fio.unicen.edu.ar (V.F.R.); mtrezza@fio.unicen.edu.ar (M.A.T.); atironi@fio.unicen.edu.ar (A.T.); ccastellano@fio.unicen.edu.ar (C.C.C.); firassar@fio.unicen.edu.ar (E.F.I.); 2 Department of Materials Engineering and Chemistry, Faculty of Civil Engineering, Czech Technical University in Prague, Thákurova 7, CZ-166 29 Prague, Czech Republic; milena.pavlikova@fsv.cvut.cz (M.P.); jaroslav.pokorny@fsv.cvut.cz (J.P.); 3 Department of Inorganic Chemistry, Faculty of Chemical Technology, University of Chemistry and Technology, Technická 5; 166 28 Prague 6, Czech Republic; ondrej.jankovsky@vscht.cz

**Keywords:** pozzolan, red ceramic waste, heat of hydration, analysis of hydrated products, physical and chemical parameters

## Abstract

Two waste fired brick powders coming from brick factories located in Argentine and Czech Republic were examined as alternative mineral admixtures for the production of blended cements. In pastes composition, local Portland cements (Argentine and Czech) were substituted with 8–40%, by mass, with powdered ceramic waste. For the ceramic waste-Portland cement system, workability, the heat released, pozzolanity, specific density, compressive strength, hydrated phases, porosity, and pore size distribution were tested. The relevance of the dilution effect, filler effect, and pozzolanic activity was analyzed to describe the general behavior of the pozzolan/cement system. The properties and performance of cement blends made with finely ground brick powder depended on the composition of ceramic waste and its reactivity, the plain cement used, and the replacement level. Results showed that the initial mini-slump was not affected by a low ceramic waste replacement (8% and 16%), and then it was decreased with an increase in the ceramic waste content. Brick powder behaved as a filler at early ages, but when the hydration proceeded, its pozzolanic activity consumed partially the calcium hydroxide and promoted the formation of hydrated calcium aluminates depending on the age and present carbonates. Finally, blended cements with fired brick powder had low compressive strength at early ages but comparable strength-class at later age.

## 1. Introduction

The environmental concern and energy consumption are currently two major topics in the manufacturing of cement-based materials. The incorporation of mixed construction and demolition wastes as a substitute for natural aggregates has many economic and environmental benefits [1,2,3,4]. Large amounts of fuels are consumed during the process of cement production, which makes the cement industry also contribute to the rising of atmospheric CO_2_ concentration. According to the available statistics, the amount of CO_2_ emission for the process of cement production occupies 8–9% of the global anthropogenic CO_2_ emissions and 2–3% of energy use [5]. Therefore, a great deal of efforts has been made in previous studies to identify alternative or supplementary cementitious materials (SCMs) to fully or partially replace Portland cement (PC) in concrete. Within the feasible alternative materials for PC replacement, natural materials, less embodied-energy materials, industrial wastes, or by-products from different manufacturing processes having pozzolanic properties, such as volcanic rocks [6], coal fly ash and bottom ash [7,8,9,10], zeolite [11,12], biomass-based ashes [13,14,15,16], ground granulated blast-furnace slag [17], waste glass powders [18], and many others [19,20], exist.

Recently, studies on possible use of recycled powders produced from construction and demolition waste as SCMs have received a great attention of materials researchers due to the management of the increasing amount of demolition waste generated at the end-of-life of structures [21], whereas the fine ceramic dust obtained during the grinding and polishing of ceramic products is also available [22]. However, despite the abundance of construction and demolition waste, the applications of fine particles as PC substitute are currently very limited [23] and have not been adequately investigated when compared to larger particles used as concrete fine and coarse aggregate [24,25].

For the use of the ceramic wastes as SCMs, that is, as a part of blended binder, the changes in composition fluctuations and raw materials must be taken into account [26,27]. However, not all modern bricks have pozzolanic properties. Some are fired in a kiln at high temperature but can have a low content of clayed minerals [28]. Then, the ceramic wastes from different manufacturers that have the same appearance may differ in their composition depending upon the geological origin of raw materials and the thermal process used.

Several researchers have proposed alternatives in order to use the ceramic wastes, particularly roof tiles [29,30,31,32,33], bricks [24,34,35,36,37,38,39], and floor tiles [40]. Sabir et al. [41] found that the partial replacement of PC in the concrete mix by ground red brick caused its low early compressive strength, but the later strength (90 days) was similar to or greater than that of the PC-based concrete. Pacheco-Torgal and Jalali [42] found that 20% of PC substitution with ground red brick (w/b = 0.6) gave concrete low strength loss and increased its durable performance. The Brazilian researchers estimated that a 10% reduction of CO_2_ emissions could be obtained by replacing 20% of the cement with CW (ceramic waste) [43]. Recycled clay-brick-powder and fly ash were also used as partial substitutes of Portland cement in the self-consolidating concrete mix [44]. Bektas et al. [45] reported that 25% PC replacement by ground brick in the concrete mix had no significant effect on the water demand, but the hardened materials had low compressive strength at early ages and comparable strength to PC-concrete at later ages. Moreover, ground brick improved the chloride ion penetration resistance of concrete. A previous study [46] with the replacements of up to 60% of PC with ground CW suggested that over 20% of CW acted as a filler. Blended cements up to 35% in mass of ground tile wastes accomplish with the physical requirements of Turkish standard [40], which indicates the possible wide application of this practice.

Considering the previous results reported in papers, the aim of this research was to analyze the interaction between ceramic wastes and Portland cement, independently of composition, source, and changes in raw materials, in order to understand the universal behavior of CW-PC system for industrial application. Two ceramic wastes with different characteristics by origin (Argentine and Czech Republic) were used in PC blends with wide replacement percentages (from 0% to 40%). The CW-PC system was fully characterized, and the relevance of dilution effect, filler effect, and pozzolanic activity was analyzed to describe the universal behavior of the system. Based on literature analysis, such complete experimental campaign was not presented yet as most of the papers aimed at using CW dust as PC substitute on the basis of its contradictory contribution to the mechanical resistance, durability, and workability only. On the other hand, complex studies of the time development of hydrated products and physical parameters of CW-PC system were only scantly presented up to now, although they represent quite unique information on the behavior of CW powder in PC blend.

## 2. Materials and Methods

### 2.1. Used Materials and Their Characterization

In this cooperative research, two sets of materials were studied to make the same test-plan. One set was a Portland Cement CEM I and ceramic waste from Olavaria city, Argentine (ArgPC, ArgCW), and the other set was Portland Cement CEM I and ceramic waste from Olavaria city, Czech Republic (CzPC, CzCW). The cements used were tested as delivered from cement factories. The ArgCW consisted of a scrap discarded as waste in a local brick manufacturer at LOIMAR Tandil Plant. In this factory, clay bricks are calcined at about 950–1050 °C. The ArgCW was crushed and finely ground in a laboratory ball grinding mill of the cement manufacture plant. The CzCW was a by-product originating from the HELUZ Brick Industry, a major brick producer on the Czech market, at the Hevlín production plant. It was obtained during the grinding of highly precise cavity brick blocks previously fired at about 800–850 °C. The CzCW was used as collected in the factory.

#### 2.1.1. Chemical Characteristics

Table 1 reports the chemical and mineralogical composition of both cements and CWs determined by X-ray fluorescence (XRF) and X-ray diffraction (XRD). XRF analysis of cements and CWs was performed using an Axios sequential WD-XRF spectrometer (PANalytical, Almelo, The Netherlands) equipped with an Rh anode end-window X-ray tube fitted with a 50 μm beryllium window. The resulting data were collected by SuperQ software (v 1.2) and further evaluated by Omnian software (v 1.2) and further evaluated by Omnian software (v 1.2). As the content of SiO_2_ + Al_2_O_3_ + Fe_2_O_3_ was higher than of 70% by mass and the loss on ignition (LOI) was lower than 6%, both CWs met the requirements of the specifications of ASTM C 618 for calcined pozzolan addition [47].

The mineralogical composition of both binder constituents was determined by X-ray diffraction (XRD) analysis using Philips PW 3710 diffractometer (Phillips, Netherland) operating with CuKα radiation at 40 kV and 20 mA using carbon monochromator. XRD data showed that ArgPC had a low C_3_A content (~2.5%) and the CzPC had a medium C_3_A content (~6.5%). Both PCs had calcite as a minor component (<5%). Sulfate supplying phase was predominately gypsum for CzPC, and it was composed of gypsum, basanite, and arkanite in the ArgPC.

**Figure 1:**
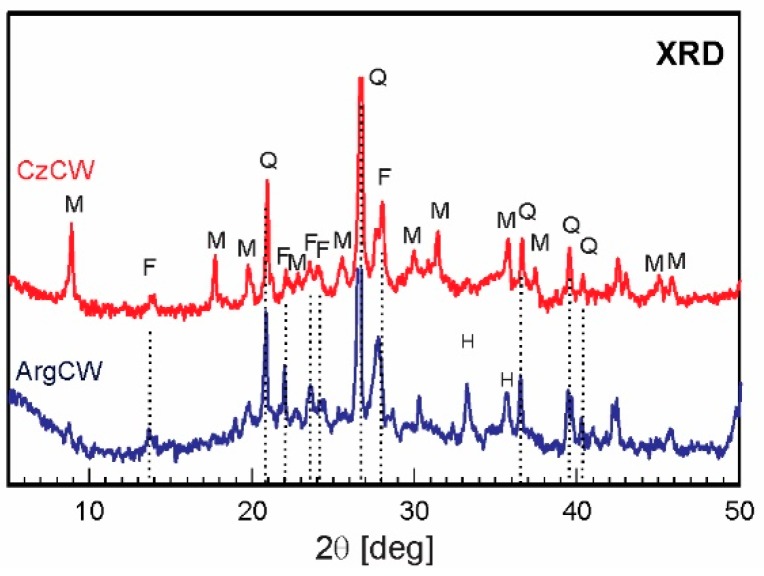
XRD pattern of ceramic waste used (M = muscovite; F = Feldspar; Q = Quartz; H = Hematite).

XRD patterns of studied ceramic powders are shown in Figure 1. The identified crystalline compounds were quartz, feldspars (mainly as anorthite), and low content of hematite for ArgCW; and quartz, feldspars (albite, microcline, and orthoclase), and mica (muscovite and biotite) for CzCW. The glassy phase content (dome from 2θ = 18° to 2θ = 38°) was calculated using Rietveld analysis with internal standard (ZnO). The content of the glassy phase was 37.3 wt.% and 46.7 wt.% for ArgCW and CzCW, respectively. As the content of the glassy phase was higher for CzCW than that of ArCW, we analyzed its composition in detail. The main compounds forming glassy phase of CzCW were estimated as follows (in wt.%): SiO_2_ 24.42, Al_2_O_3_ 33.73, Fe_2_O_3_ 7.06, CaO 18.96, MgO 7.94, SO_3_ 1.2, K_2_O 2.29, TiO_2_ 1.49.

#### 2.1.2. Physical Characteristics

The PCs and CWs density measured on a helium pycnometry principle (Pycnomatic ATC, Porotec GmbH, Thermo Electron Corporation, Hofheim/ts., FRG), and the Blaine specific surface area (EN 196–6 [48]) and the particle size distribution (PSD) measured by a laser diffraction method with a dry dispersion (Mastersizer 2000, Malvern Panalytical, Malvern, UK) are also reported in Table 1. As it is a generally accepted material, it itself can potentially exhibit a pozzolanic activity, but if the particles are too large, they cannot be utilized effectively, and the pozzolanic reaction is hindered or too slow to contribute to the final properties of the hydrates system. Data given in Table 1 for PCs confirmed their CEM I 42.5R and CEM I 32.5 classes. The CzCW was slightly finer than ArgCW; nevertheless, both tested powders met criteria of ASTM C 618 [47] and EN 450-1 [49] that set upper limits for the particle size of pozzolans. The limit for pozzolan particle size set in EN 450-1 was 45 μm in the form of 40% residue on the 45 μm sieve, and in ASTM C618, the limit was 34% on the same sieve. The water absorption measured for samples immersed for 24 h in water as prescribed in EN 1097-6 [50] was much greater for ArCW than that of CZCW (see Table 1). Although CzPC was the finest material, its Blaine specific surface was much lower compared to that obtained for ArCW and CzCW. It was due to the porous character of CW particles indicated by the water absorption data and morphology scans. Then, the Blaine test based on air permeability method identified both the external and internal surfaces of particles.

**Figure 2:**
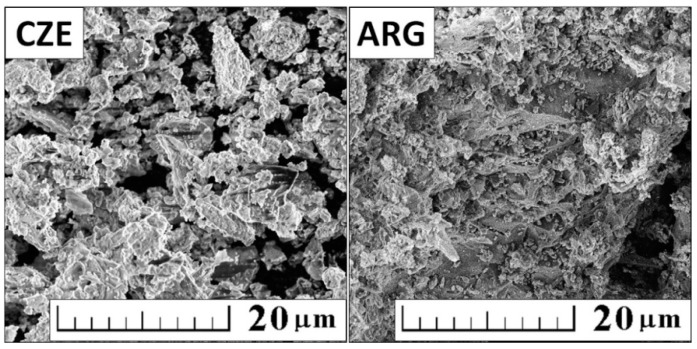
Morphology of ceramic powders obtained by SEM.

The morphology of CWs was examined using a scanning electron microscope (SEM, TESCAN Lyra 3, Tescan Brno, s.r.o., Brno, Czech Republic) equipped with a Field Emission Gun (FEG[S3]) electron source. Elemental composition and mapping were performed using energy dispersive spectroscopy (EDS) analyzer (XMaxN) with a 20 mm^2^ SDD detector (Oxford Instruments, High Wycombe, UK) and AZtecEnergy software (v. 4.0 sp2). To conduct the measurements, the samples were placed on a carbon conductive tape and sputtered with 10 nm of gold. SEM and EDS measurements were carried out using a 10 kV electron beam. The morphology of analyzed ceramic powders is shown in Figure 2. Angular-shaped particle morphology was observed in small quantities. Besides, CW particles mostly had an irregular shape and partially layered microstructure with a high percentage of finer materials (most of the particles less than 15 µm for CzCW and 20 µm for ArgCW). According to SEM, obtained particles were slightly smaller in comparison to PSD, probably due to the presence of some agglomerates.

**Figure 3:**
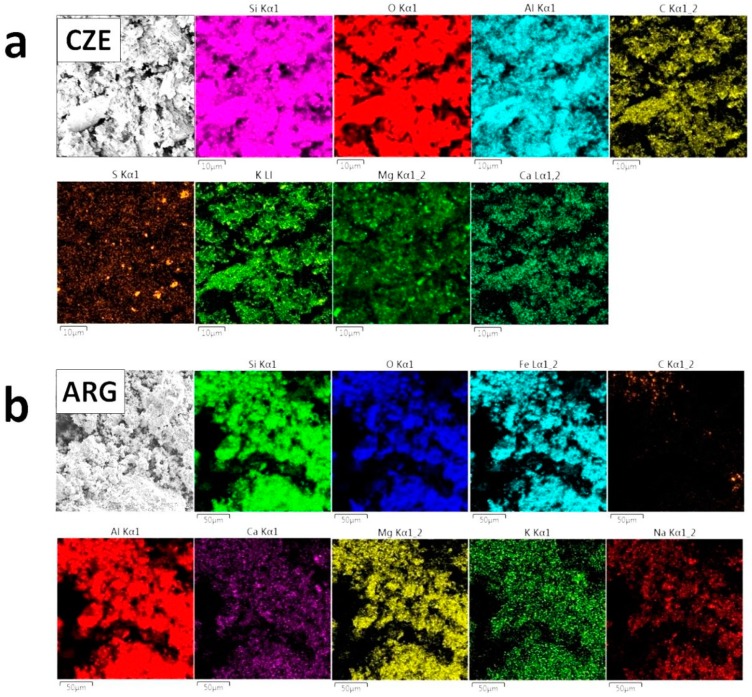
Elemental distribution maps of the major elements in ArgCW (**a**) and CzCW (**b**) obtained by energy dispersive spectroscopy (EDS). Scale bar is 10 μm and 50 μm, respectively.

The elemental distribution maps analyzed by energy dispersive spectroscopy (EDS) are shown in Figure 3. Except for oxygen, high content of silicon, alumium, and iron was confirmed in ArgCW. In CzCW, silicon, calcium, aluminum, and magnesium prevailed, which was in agreement with XRF data and EDS results reported by Subaşi et al. [51]. Also, the presence of sodium was detected in ArgCW, similarly as by XRF. On the other hand, sulfur was found in CzCW. In both samples, the presence of potassium was confirmed. All the identified elements were in the tested specimens distributed uniformly. In this respect, it should be mentioned that the composition obtained by EDS is not shown here. While XRF is a much more precise method, the obtained values are referred to a much larger area and also the sensitivity of XRF is higher.

#### 2.1.3. Mix Proportion and Preparation

Blended cements (bc) were prepared by replacing 8% to 40%, by mass, of PC with CW and were labeled as the percentage and source of CW (see Table 2). The water/binder (w/b) ratio for all PC/CW blends was 0.5. The pastes were mixed in planetary laboratory machine and were remixed every 15 min until 1 h before the initial setting time to avoid the paste segregation before testing or casting.

### 2.2. Methods of Testing Fresh and Hardened Pastes

For all blended cements samples, the mini-slump test, the pozzolanic activity, the heat released, the compressive strength, the specific density, and the pore size distribution were determined. In addition, the identification of hydrated products was done by XRD.

#### 2.2.1. Mini-Slump Test

To assess the paste workability, the mini-slump test was used [52]. Immediately after mixing, the cone resting on a glass plate was filled with the cement paste. The cone was gradually lifted to allow the flow of paste on the glass, and after one minute, two orthogonal spreading diameters of the pad were measured and the average was calculated. The remaining paste in the bowls was remixed every 15 min to avoid the bleeding, and the mini-slump was measured at 30, 60, 120, and 180 min.

#### 2.2.2. Frattini Test

Pozzolanic activity of blended cements was determined by Frattini test in compliance with EN 196-5 [53]. In this method, 20 g of blended cement was mixed with 100 mL of boiled distilled water. After preparation, samples were left for 2, 7, and 28 days in a sealed plastic container at 40 °C. During the test time, the samples were vacuum filtered through the paper in sealed kitasato flask. The filtrate was analyzed for [OH^−^] by titration against dilute HCl with methyl orange indicator and for [Ca^2+^] by pH adjustment to 13, followed by titration with 0.025 M EDTA (11.17 g of ethylendiaminetetraacetic acid in to 1 L of distilled water) solution using Murexide indicator. This test compares the [Ca^2+^] (expressed as [CaO]) and [OH^−^] contained in an aqueous solution that covers the hydrated sample with the solubility curve for CH in an alkaline solution at the same temperature. The blended cement is considered as pozzolanic when the calcium hydroxide concentration in the sample solution is located below the solubility isotherm of calcium hydroxide.

#### 2.2.3. The Heat of Hydration Measurement

The rate of heat evolution and the cumulative heat released during hydration were measured under isothermal conditions at 20 °C in conduction calorimeter. The paste was prepared using 20 g of cementitious material in a small plastic bag carefully homogenized. The bag and mixing water were placed into the calorimeter until the thermal stabilization, and then the cement was mixed with 10 g of water by hand for 30 s. The bag was sealed, carefully placed in the calorimetric cup, and the measurement started immediately. The hydration of paste was monitored for 48 h.

#### 2.2.4. Specific Density

For 0, 8, 24, and 40% CW and cements blends, the specific density of the pastes was measured on a helium pycnometry principle using Pycnomatic ATC (Porotec GmbH, Thermo Electron Corporation, Hofheim/ts., FRG). The accuracy of the gas volume measurement using this device was ± 0.01% from the measured value, whereas the accuracy of the used analytical balance was ± 0.0001 g.

#### 2.2.5. Compressive Strength

The compressive strength was determined on paste prism specimens with a transversal section of 1.6 × 10^−3^ m^2^. The test was conducted according to EN 196-1 [54]. Specimens were cured during 24 h in molds and later stored in the laboratory (temperature 21 ± 2 °C, RH 50 ± 5%) in sealing condition until test age: 2, 7, 28, and 90 days. At the test age, the specimens were tested using a standard compression jig, and the reported value of compressive strength is the mean of four tests. The relative expanded uncertainty of the compressive strength test was 1.4%.

#### 2.2.6. Hydration Monitoring

To study the progress of hydration, blended cement pastes were prepared and cured in sealed plastic bags at (21 ± 2) °C for 2, 7, 28, and 90 days. At this time, fragments of paste samples were carefully ground to particle size lower than 45 µm, and the crystalline hydration phases were identified by XRD analysis. The determination was performed on Philips PW 3710 diffractometer operating with CuKα radiation at 40 kV and 20 mA using carbon monochromator. The scan speed of 2 °/min and the sampling interval of 0.02° (2θ) were used.

#### 2.2.7. Pore Size Distribution

The pore size distribution of tested pastes was measured on a mercury porosimetry principle using devices Pascal 140 and Pascal 440 (Thermo Scientific, Milano, Italy). Before the measurement, the particular samples were dried in a vacuum drier. The sample mass was typically about 1 g. Within the evaluation of measured data, the circular cross-section of pores was assumed. The following parameters were used for the calculation of pore size distribution: Hg density = 13.5414 g/cm^3^ at 22 °C, capillary radius = 1.5 mm, Hg contact angle = 130 °C, Hg surface tension 480 dyne/cm.

## 3. Results and Discussion

### 3.1. Workability

**Figure 4:**
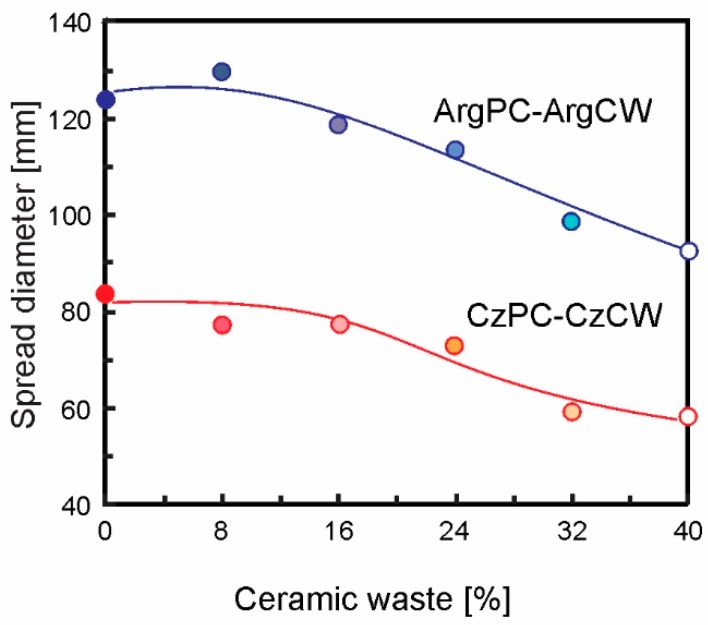
Results of the mini-slump test expressed as initial spread diameter of cement pastes containing different amounts of ceramic waste (CW).

Figure 4 shows the fluidity of the cement pastes containing different amounts of CW expressed as the initial spread diameter measured at 5 min after mixing.

It can be observed that for the ArgPC paste, an initial spread diameter (124 mm) was higher than the corresponding CzPC (84 mm). It was caused by the different mineralogical composition (medium C_3_A) and the high specific surface area of CzPC, that increased the water demand to react and produce the early hydrated products and to wet its surface area [55]. When the CW replacement increased from 0% to 40% (Figure 4), the initial spread diameter decreased from 124 to 92 mm and from 84 to 58 mm for ArgPC-ArgCW and CzPC-CzCW, respectively. For both CW-PC systems, the initial flowability decreased with increasing CW content, with the exception of 8% blend substituted with ArgCW. Compared with the respective PC, the initial mini-slump reduction was lesser than 10% and greater than 25% for low (8 and 16%) and high (40%) replacements, respectively. After 30 min, the mini-slump loss had a similar rate to the respective PC. Up to 180 min, the mini-slump loss rate was 0.22–0.25 mm/min and 0.07–0.10 mm/min for ArgPC-ArgCW and CzPC-CzCW blended cements, respectively [56].

With respect to water demand, the results of mini-slump showed that up to 24 wt.% of CW in cement blend did not cause a significant increase in water demand. Bektas et al. [34] found that the high water absorption capacity of the CW used as fine aggregate affected the mortar flow. But, when it was ground to fine particle size, the water absorption of CW was significantly reduced. For a high CW replacement, the loss of initial mini-slump was attributed to the water demand caused by the changes in the packing of blended cements due to the low density of CW that increased the solid volume. As CW-water-absorption was low, the mini-slump loss rate was similar to that of the corresponding PC.

### 3.2. Pozzolanic Activity

**Figure 5:**
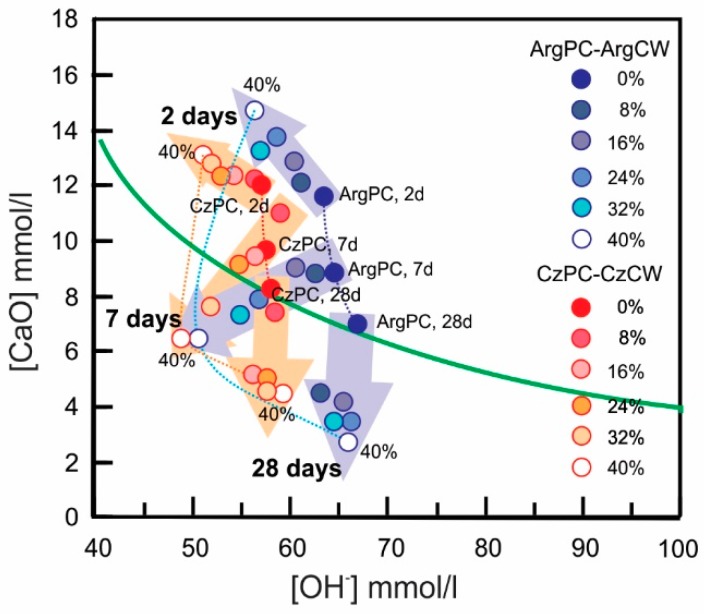
Results of Frattini test on day 2, 7, and 28.

Figure 5 shows the results of Frattini test.

At day 2, for both PC-CW combinations, all measured points were above the calcium solubility isotherm, revealing that all blended cements did not have pozzolanic activity. At day 7, the blended cements containing low replacement level (8 and 16%) did not have pozzolanic activity, and the blended cements containing 24% were located on the calcium isotherm curve for both combinations. For high replacement levels (32% and 40%), all points dropped below the solubility isotherm curve indicating that the pozzolanic material reacted with dissolved calcium hydroxide to high rate giving an unsaturated supernatant solution. At day 28, all blended cements appeared to have good pozzolanic activity. This test determined that both CWs had good pozzolanic activity and they could be classified as slow reactive pozzolanic materials, such as low calcium fly ash.

### 3.3. Hydration Heat

**Figure 6:**
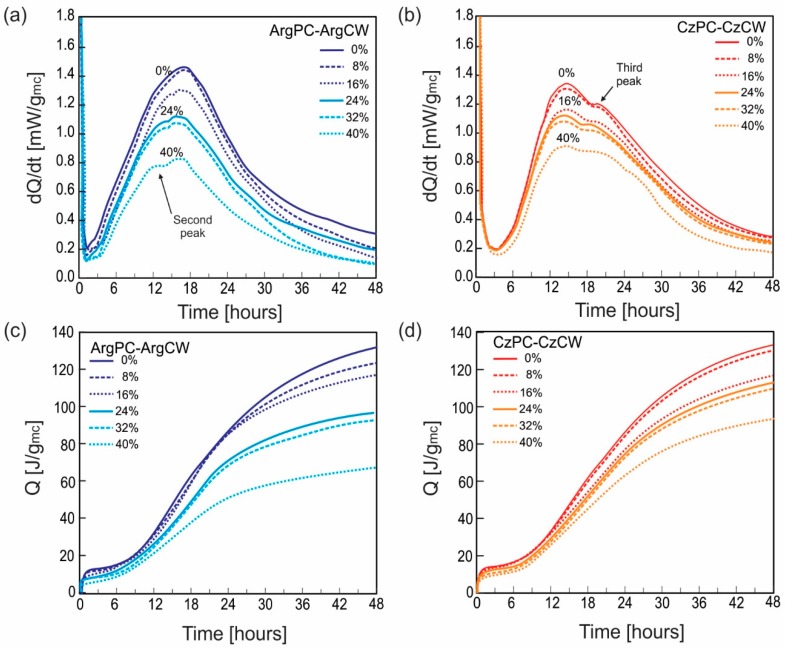
The heat of evolution and cumulative heat released for blended cements: (**a**) ArgPC-ArgCW; (**b**) CzPC-CzCW.

Figure 6 shows the results of the rate of heat evolution (mW/g) and cumulative heat released (J/g) for both systems. The heat of evolution and cumulative heat released were normalized to g of the binder. The heat released curve for blended cements up to 48 h can be divided into five periods: (1) dissolution and wetting with a high exothermic signal during the first 15 min; (2) the dormant period was characterized by the duration first valley; (3) the acceleration period corresponding to the C_3_S hydration that caused the second peak characterized by the slope, the maximum value of the rate of heat released, and the time when maximum signal occurred; (4) the third peak was associated with the C_3_A hydration and characterized by its intensity and time of occurrence; (5) the deceleration period where the thermal signal decreased slowly, and no singular points were detected. The values of the characteristic points of the heat released curve and the cumulative heat released (J/g) are reported in Table 3, where Cmax2 [mW/g] means the maximum value rate of the second peak of heat released curve with tmax2 [min] the corresponding time and Cmax3 [mW/g] is the maximum value rate of the third peak detected at time tmax3 [min].

Analyzing the curve of the rate of heat evolution for both PCs, it could be observed that the dormant period was shorter in the ArgPC (~2 h) than that of CzPC (~3 h). After the dormant period, the intensity of the second peak was higher for ArgPC at the same occurrence time (~14 h). For CzPC, its moderate C_3_A content produced the third peak ~4.5 h after the second peak, which was not revealed in pastes with the ArgPC (low C_3_A). Finally, the descending branch during the deceleration period was more pronounced for ArgPC. From the cumulative heat of hydration data, both PCs had similar value after 12 h up to 48 h. The early difference occurred due to the greater amount of C_3_S in ArgPC, which caused a more pronounced acceleration period. For both PCs, the increase in CW replacement presented the same response on the curve of heat evolution up to 48 h. Blended cements with CW copied the shape of the dQ/dt curve of the corresponding PC without significant variation in the time of occurrence for the characteristic peaks and with a declined intensity of the heat signal with an increased replacement level.

For both blended cements with 8% of CW, the heat released curves were practically the same as those of the corresponding cement and their parameters (slope of acceleration, intensity, and time of occurrence of second and third peaks) and had similar values. The deceleration slope had a high value until 24 h. The cumulative heat of blended cements with 8% CW was in the same value range as the corresponding PC. This behavior was attributed to the stimulation effect caused by the CW incorporated to blended cement that compensated the dilution effect. The stimulation effect was observed in the Frattini test results on day 2 (Figure 5). When the ratio of PC replacement was increased, the content of [CaO] and [OH^−^] increased and decreased, respectively, due to the proper PC hydration.

When the CW replacement level increased from 16 to 32%, the heat released rate up to 48 h was reduced proportionally to the CW replacement level. For the ArgPC-ArgCW blended cements, the acceleration slope and the intensity of the second peak were reduced as occurred also for CzPC-CzCW blended cements. But the time of occurrence remained constant, revealing that CW acted without interference into the cement hydration.

For the CzPC-CzCW, the intensity of the third peak also decreased with an increase in the CW replacement level, but it can be observed that the ratio between the intensity of the second peak and third peak increased, indicating that CW addition stimulated the alumina phase of cement hydration. The cumulative heat was lower than the corresponding cement at all testing ages. For 40% PC substitution with CW, the duration of the dormant period was longer, and the heat released and the cumulative heat considerably declined.

### 3.4. Specific Density and Porosity

Table 4 reports the evolution of the specific density of examined pastes measured for the specimens for 2, 7, and 28 days. The density of the paste depends on the assemblage of not hydrated and hydrated compounds and the porosity. For all pastes, the specific density drops between 2 and 7 days due to the progress of cement hydration and later it has a small change between 7 and 28 days. For ArgPC, the density of blended cements was higher than the plain cement. CzPC and its blended cements had low-density matrix at all ages, and it was attributable to rapid hydration of this cement and the large volume of ettringite [56].

**Figure 7:**
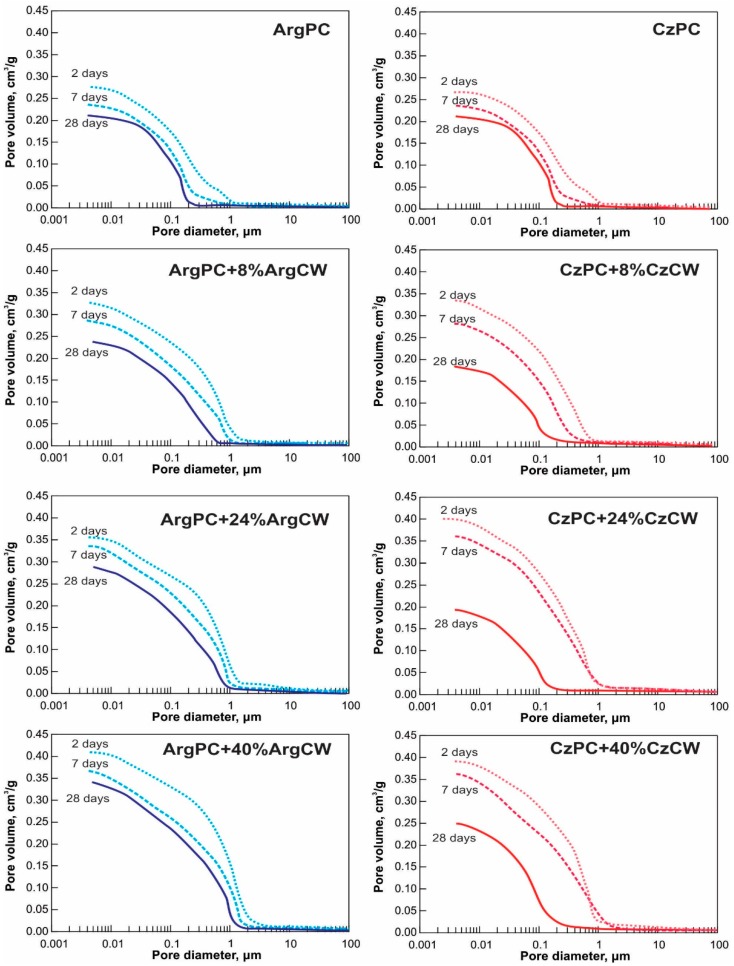
The Pore size distribution of blended pastes.

Table 4 also reports the results of mercury intrusion porosimetry (MIP) parameters assessed for pastes containing 0, 8, 24, and 40 wt.% of CW aged 2, 7, and 28 days. Additionally, the pore size distribution curves are also introduced, as shown in Figure 7. As expected, the increased curing time reduced the total cumulative pore volume and decreased the pore threshold diameter for all blended cements due to hydration progress. Compared with the corresponding PC at the same age, the increasing percentage of CW increased the total cumulative pore volume and the pore threshold diameter for blended cement pastes in accordance with results obtained by O’Farrel et al. [57].

For both PC pastes, the decrease of total pore volume with the time occurred principally for the pore diameter larger than 0.05 µm, while the volume of pores smaller than 0.05 µm remained approximately constant. At day 2, the threshold pore diameter of CzPC was smaller compared to that of corresponding ArgPC, and later, both pastes had a similar value.

Compared with the ArgPC paste, the incorporation of 8 wt.% to 40 wt.% of ArgCW increased the total pore volume of the blended cement pastes from 20% to 50% and 22% to 57% at day 2 and 7, respectively. The main difference was the increase in the volume of pores lower than 0.05 µm on day 7 with an increase in the CW replacement. For 0 wt.% to 40 wt.% of CW in pastes, the threshold diameter increased from 1.126 to 2.641 µm and 0.224 to 1.747 µm at day 2 and 7, respectively. At day 28, the total pore volume was larger than that of plain cement paste.

For CzCW-CzPC, the total cumulative pore volume for all blended cement was similar to that of ArgPC-ArgCW on day 2 and 7. But, it was very low for the samples at day 28. For 8% and 24% cement replacements, it was even lower than that of corresponding CzPC paste, but for 40% PC replacement, it was still larger than that of the PC paste. From 7 to 28 days, the change in the threshold pore width (0.36 to 0.16 and 0.97 to 0.18 µm) in blended cement containing 8 wt.% and 24 wt.% of CzCW gave evidence on the progress of hydration producing the pore size refinement due to coarse pores filling, and the threshold pore diameter was reduced to a size comparable with the PC paste. Regarding the threshold pore diameter, it is proportional to CW replacement at day 2 and 7. But, it was clearly reduced to a smaller diameter than CzPC paste for all replacements on day 28.

### 3.5. Compressive Strength of Hardened Pastes

**Figure 8:**
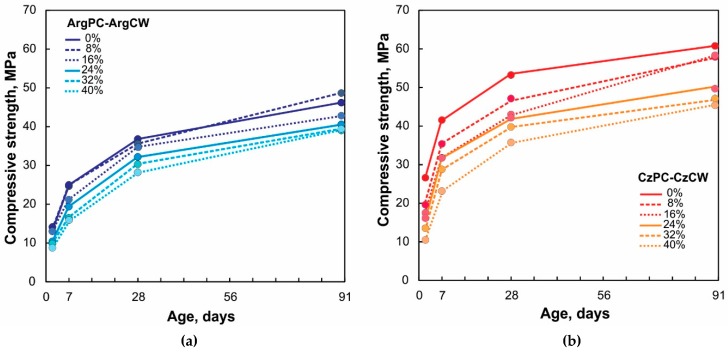
Compressive strength of blended cements: (**a**) ArgCW-ArgPC and (**b**) CzCW-CzPC.

Figure 8 shows the compressive strength development of pastes containing different amounts of CW for both PC combinations. For all ages of the studied samples, the strength of CzPC was greater than the corresponding ArgPC. For ArgPC-ArgCW, PC could be replaced with ground CW up to 16 wt.% without impairing the compressive strength at day 28. Replacing 8% PC by CW even retained or increased the compressive strength at all ages. From 7 to 28 days, the strength gain in pastes with CW was attributed to the progress of the pozzolanic reaction. On day 90, the compressive strength of blended cement with 8 wt.% of CW was greater than the corresponding ArgPC. The ratio between compressive strength of the blended cement and PC was higher than 0.75 for blended cements containing 8, 16, and 24 wt.% of ArgCW from 2 days to 90 days. For 32% and 40% ArgPC replacement, it requires 28 days to attain this ratio.

For CzPC-CzCW, the compressive strength of blended cement was reduced with the CW replacement from 2 to 28 days. On day 90, blended cements containing up to 16 wt.% of CW had a similar strength to CzPC. The strength ratio was higher than 0.75 on day 2, 7, and 28 for low (8%), medium (16% and 24%), and high (32%) cement replacements, respectively. Blended cements with 40 wt.% of CW attained this ratio on day 90.

The less reduction of compressive strength of blended cements with low CW-content was attributed to the high hydration degree of PC phases that compensated the dilution effect. When the CW replacement level increased (>16%), the dilution effect predominated over the stimulation effect, decreasing the heat released and compressive strength.

**Figure 9:**
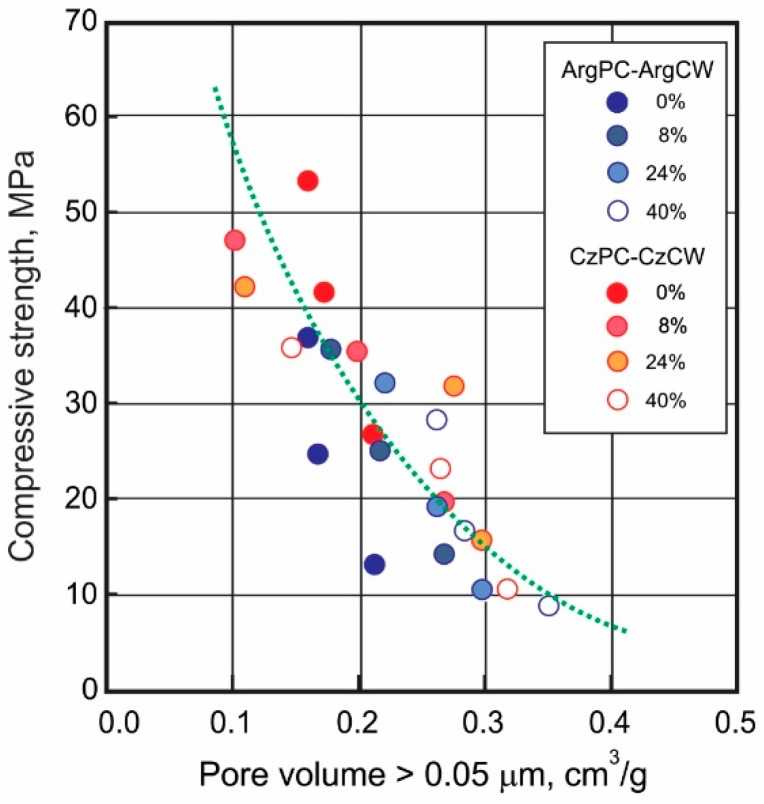
Relationship between the cumulative pore volume of pores greater than 0.05 µm and the compressive strength.

According to Mindess and Young [58], the compressive strength was related to the total volume of the pore with a diameter greater than 0.05 µm. Figure 9 shows the relationship between the compressive strength and the volume pore (diameter > 0.05 µm). At an early age, the compressive strength decreased due to the high porosity of blended cement with high CW content. The 28 days’ strength increased due to the later reduction of pore volume caused by the progress of the pozzolanic reaction of CW.

### 3.6. Kinetics of Pozzolanic Reaction and Formed Compounds

**Figure 10:**
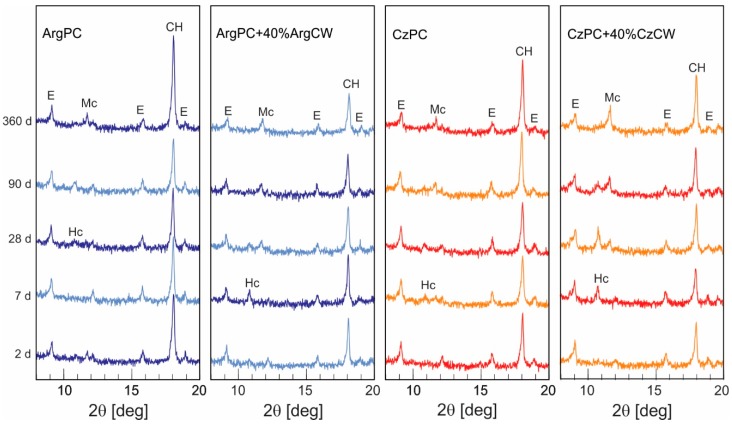
XRD pattern for (**a**) ArgPC; (**b**) ArgPC + 40%ArgCW; (**c**) CzPC; (**d**) CzPC + 40%CzCW. (E: ettringite, Hc: hemicarboaluminate, Mc: monocarboaluminate, CH: calcium hydroxide).

The hydrated crystalline phases and their relative main peak intensity identified by XRD are summarized in Table 5. Complementarily, Figure 10 illustrates the XRD patterns from anhydrous phases up to 360 days of hydration for both PCs and blended cements with 40 wt.% of CW.

In both reference PC pastes, on day 2, ettringite (E) and calcium hydroxide (CH) were identified. AFm phases were absent, and some cement phases (C_4_AF and C_2_S) remained. For all blended cements with ArgCW and CzWC (Figure 6b,d), the crystalline hydrated products detected were E and CH as occurred in both PCs.

On day 7, the E and CH were accompanied by the formation of AFm phase assigned as hemicarboaluminate (Hc) in both PCs. For blended cements, the pozzolanic reaction between CW and CH produced a cementing compound like C–S–H and alumina phases that depended on the available CH and the CW reactivity. When available, CH and the alumina reactive contents in CW were high in free carbonate sample, and the crystalline hydrated phase obtained was C_4_AH_13_, but in the presence of small carbonate proportion (<3%) [59], its transformation to Hc (C_3_A∙CH_0.5_ CC_0.5_∙H_12_) occurred. This crystalline phase was associated with the pozzolanic reaction, and its main peak had a greater intensity for blended cements with 40 wt.% of CW.

On day 28, for blended cements, the intensity of CH peak decreased, and the formation of monocarboaluminate (Mc) was incipient, and it coexisted with the presence of Hc. The intensity of the peaks assigned to Hc and Mc on day 28 was greatest in CzPC-CzCW. According to XRD data, the AFm phases increased, and the transformations of Hc to Mc occurred when the calcite reacted [60]. This transformation was more intense in the CzPC having large C_3_A content. The reactive alumina provided by CW was stabilized as Mc in this system due to the fact that its low solubility product was constant (logK_sp_ = –25.4, –29.75, and –31.47 for C_4_AH_13_, Hc, and Mc, respectively) [61]. Finally, the Mc was a well-defined hydrated product on day 90 (Figure 10), and its peak intensities increased with the PC replacement ratio (Table 3). This gave evidence of pozzolanic reaction, which, however, could not compensate for the dilution effect, and the total pore volume of blended cement was larger than the corresponding PC. Another indication that the pozzolanic reaction started was the increase in the volume of pores with a diameter lower than 0.05 µm, which was attributed to this reaction. On day 360, the Mc was the AFm present in all blended cements. The pozzolanic reaction of CW was limited, and the CH was clearly detected in all blended cements on day 360.

The incorporation of CW to blended cement changed the cement hydration due to: the effect of particle size distribution (filler effect), the heterogeneous nucleation (stimulation of the hydration of PC), the dilution effect (increases of effective w/c), and the pozzolanic reaction that produced cementing compounds at a different rate than PC [62]. When the dilution effect was greater than the compensating effects (filler, stimulation, and pozzolanic), a smaller amount of hydrated phases were found in the system, causing a large volume of pores. On the other hand, when the volume of hydration products caused by the stimulation and the pozzolanic reaction compensated or overpassed the effect of dilution, the volume of hydrated phases caused a smaller volume of pores.

## 4. Conclusions

The performance of blended cement made with finely ground CW depends on the composition of CW and its reactivity, the plain cement used, and the replacement level. These variables determine the development of the hydration reaction that produces the cementing compounds and reduces the porosity of paste. Based on the results of this study, the following conclusions can be generalized for the PC-CW system:

- Ceramic waste originated in a ceramic factory can be used as a pozzolanic material contributing to the reduction of CO_2_ emission in cement-based materials.
- The initial mini-slump was not affected for low PC replacement (8% and 16% by mass) but decreased with increasing CW content. The mini-slump loss rate of blended cement had similar value to the corresponding PC.
- The incorporation of CW reduced the heat released during cement hydration up to 48 h without significant changes in the time of occurrence of main hydration peaks.
- CW behaved as a filler at early ages, but when the hydration proceeds, its pozzolanic activity consumed partially the CH and promoted the formation of Afm phases depending on the age and carbonates present. On day 7, the formation of hemicarboaluminate occurred, then it coexisted with the incipient formation of monocarboaluminate, and finally, the monocarboaluminate was the predominant Afm phase.
- Blended cements with CW had low compressive strength at early ages but comparable strength-class at later age. Blended cement pastes containing CW exhibited higher porosity than those of PC paste. However, a reduction in the threshold value for more reactive CW was observed.
- Based on proven pozzolanic activity and low-density of studied powdered red ceramic wastes, their use in mix composition of cement and lime-based composites can be anticipated. The results summarized above indicated that CW could act both as an active mineral admixture and lightweight microfiller for the production of lightweight construction materials.

## Figures and Tables

**Table 1:** Chemical and physical characteristics of used materials.

Element/Compound	Portland Cement		Waste Ceramic Powder	
	ArgPC	CzPC	ArgCW	CzCW
**Chemical Composition, wt.%**				
SiO_2_	21.5	18.9	64.6	51.3
Al_2_O_3_	3.8	4.2	17	20
Fe_2_O_3_	3.8	3.8	5.6	6
CaO	64.3	62.4	2.5	11.5
MgO	0.8	1	1.5	4.5
SO_3_	2.6	2.3	-	1
Na_2_O	0.1	0.02	4.2	1.3
K_2_O	1.1	1.1	2.9	3.2
TiO_2_	-	0.8	0.7	0.8
LOI, %	2.1	1.5	0.6	1.1
**Mineralogical Composition, wt.%**				
Alite	64	67	-	-
Belite	12	8	-	-
Tricalcium Aluminate	2.5	6.5	-	-
Brownmerilleite	12.5	7.5	-	-
Calcite	4.5	4	-	-
Gypsum	2.5	6	-	-
Bassenite	1		-	-
Arkanite	0.5		-	-
Periclase	0.6	0.8		
Amorphous fraction			37.3	46.7
Quartz			30.5	23.6
Feldspar	Anhortite		28.6	
	Albite			6.5
	Microcline			7.2
	Orthoclase			2.8
Mica	Biotite		-	0.9
	Muscovite			2.6
Other silicates	Akermanite		-	4.4
	Hedenbergerite			3.8
Hematite			3.5	1.4
**Physical Properties**				
Density, g/cm^3^	3.15	3.08	2.68	2.77
SS Blaine, m^2^/kg	315	330	590	665
Water absorption, %	-	-	0.68	0.46
**Particle size distribution parameters, μm**				
d_90_	63.5	38.2	64.7	41
d_50_	19	14.2	30.2	19.5
d_10_	2.7	2.3	6	4.5

**Table 2:** Mix proportion of the analyzed pastes.

Cement Blend	Argentine		Czech	
	ArgPC	ArgCW	CzPC	CzCW
0 CW	100	0	100	0
8 CW	92	8	92	8
16 CW	84	16	84	16
24 CW	76	24	76	24
32 CW	68	32	68	32
40 CW	60	40	60	40

**Table 3:** Parameters of the heat released curve and cumulative heat released for all blended cement.

CW*%	Duration of First Valley, Min	Second Peak Parameters			Third Peak Parameters			Heat Released, J/g				
		C_max2_	t_max_	Slope	C_max3_	t_max_	C_max2_/C_max3_	6	12	24	36	48
		mW/g	min	mW/g∙s	mW/g	min		h				
**ArgPC-ArgCW**												
0	130	1.39	840	0.00170	-	-	-	10.4	31.7	88.3	117.1	125.9
8	160	1.36	849	0.00178	-	-	-	9.0	28.7	84.0	110.4	117.5
16	170	1.23	842	0.00173	1.23	1001	1.00	9.2	27.8	74.5	99.3	108.9
24	160	1.06	806	0.00151	1.10	934	1.04	9.6	26.7	69.6	91.4	97.5
32	150	1.06	800	0.00156	1.10	927	1.04	8.6	25.6	68.7	88.5	94.3
40	210	0.75	770	0.00127	0.81	920	1.08	6.2	19.0	50.3	63.5	67.2
**CzPC-CzCW**												
0	210	1.33	848	0.00264	1.19	1128	0.89	15.9	32.9	84.9	114.5	122.3
8	215	1.30	848	0.00257	1.19	1138	0.92	16.2	32.9	84.8	116.6	124.9
16	250	1.15	848	0.00238	1.07	1133	0.93	15.4	30.3	77.7	109.9	113.8
24	250	1.11	837	0.00225	1.05	1092	0.95	14.5	29.7	74.2	101.7	108.6
32	270	1.08	822	0.00228	1.02	1031	0.94	14.5	29.3	73.8	99.1	105.5
40	290	0.90	822	0.00194	0.87	1123	0.97	13.5	25.5	63.4	83.8	88.6

*CW means ceramic waste.

**Table 4:** Specific density and porosity parameters assessed by MIP*.

Age, days	CW, %	Specific Density, g/cm^3^	Cumulative Pore Volume, cm^3^/g			Threshold Pore Diameter, μm
			Total	<0.05 μm	>0.05 μm	
**ArgPC-ArgCW**						
**2**	0	2528	0.275	0.062	0.213	1.126
	8	2568	0.329	0.066	0.263	1.479
	24	2538	0.359	0.062	0.297	1.720
	40	2597	0.411	0.060	0.351	2.641
**7**	0	2342	0.234	0.066	0.168	0.224
	8	2423	0.289	0.072	0.217	0.929
	24	2420	0.342	0.079	0.262	1.168
	40	2437	0.371	0.086	0.285	1.747
**28**	0	2321	0.211	0.050	0.160	0.187
	8	2356	0.246	0.068	0.178	0.670
	24	2395	0.291	0.070	0.221	0.749
	40	2422	0.343	0.081	0.262	1.203
**CzPC-CzCW**						
**2**	0	2509	0.275	0.062	0.213	0.915
	8	2435	0.334	0.066	0.268	0.921
	24	2432	0.394	0.096	0.298	1.051
	40	2485	0.401	0.100	0.301	1.137
**7**	0	2338	0.238	0.065	0.173	0.275
	8	2297	0.284	0.085	0.199	0.364
	24	2339	0.360	0.084	0.276	0.978
	40	2407	0.366	0.101	0.265	1.156
**28**	0	2308	0.211	0.051	0.160	0.194
	8	2257	0.183	0.081	0.102	0.163
	24	2319	0.192	0.082	0.110	0.185
	40	2356	0.249	0.104	0.147	0.154

*CW means ceramic waste; MIP* is mercury intrusion porosimetry.

**Table 5:** Hydrated compound assemblage and main peak intensity determined by XRD for cement pastes.

Age, Days	Phase	ArgPC-ArgCW						CzPC-CzCW					
		0	8	16	24	32	40	0	8	16	24	32	40
2	E ^*^	✓✓ ^**^	✓✓	✓✓	✓✓	✓✓	✓✓	✓✓	✓✓	✓✓	✓✓	✓✓	✓✓
	Hc												
	Mc												
	CH	✓✓✓	✓✓✓	✓✓✓	✓✓✓	✓✓✓	✓✓	✓✓✓	✓✓✓	✓✓✓	✓✓✓	✓✓✓	✓✓
7	E	✓✓	✓✓	✓✓	✓✓	✓✓	✓✓	✓✓	✓✓	✓✓	✓✓	✓✓	✓✓
	Hc		✓	✓	✓	✓✓	✓✓	✓	✓	✓✓	✓✓	✓✓	✓✓
	Mc												
	CH	✓✓✓	✓✓✓	✓✓✓	✓✓✓	✓✓✓	✓✓	✓✓✓	✓✓✓	✓✓✓	✓✓✓	✓✓✓	✓✓
28	E	✓✓	✓✓	✓✓	✓✓	✓✓	✓✓	✓✓	✓✓	✓✓	✓✓	✓✓	✓✓
	Hc	✓	✓	✓	✓	✓	✓	✓	✓	✓	✓✓	✓✓	✓✓✓
	Mc		✓	✓	✓	✓✓	✓✓		✓	✓	✓	✓✓	✓✓
	CH	✓✓✓	✓✓✓	✓✓✓	✓✓✓	✓✓	✓✓	✓✓✓	✓✓✓	✓✓✓	✓✓✓	✓✓	✓✓
90	E	✓✓	✓✓	✓✓	✓✓	✓✓	✓✓	✓✓	✓✓	✓✓	✓✓	✓✓	✓✓
	Hc	✓	✓	✓	✓	✓		✓	✓	✓	✓	✓✓	✓✓
	Mc		✓✓	✓✓	✓✓✓	✓✓✓	✓✓✓	✓✓	✓✓	✓✓	✓✓	✓✓✓	✓✓✓
	CH	✓✓✓	✓✓✓	✓✓✓	✓✓✓	✓✓	✓✓	✓✓✓	✓✓✓	✓✓	✓✓	✓✓	✓✓
360	E	✓✓	✓✓	✓✓	✓✓	✓✓	✓✓	✓✓	✓✓	✓✓	✓✓	✓✓	✓✓
	Hc												
	Mc	✓✓	✓✓	✓✓	✓✓✓	✓✓✓	✓✓✓	✓✓	✓✓✓	✓✓✓	✓✓✓	✓✓✓	✓✓✓
	CH	✓✓✓	✓✓✓	✓✓✓	✓✓✓	✓✓	✓✓	✓✓✓	✓✓✓	✓✓	✓✓	✓✓	✓✓

* E: ettringite, Hc: hemicarboaluminate, Mc: monocarboaluminate, CH: calcium hydroxide. ** ✓✓✓ = very strong; ✓✓ = strong; ✓ = weak.
